# Microbial Diversity and Phage–Host Interactions in the Georgian Coastal Area of the Black Sea Revealed by Whole Genome Metagenomic Sequencing

**DOI:** 10.3390/md18110558

**Published:** 2020-11-14

**Authors:** Ekaterine Jaiani, Ia Kusradze, Tamar Kokashvili, Natia Geliashvili, Nino Janelidze, Adam Kotorashvili, Nato Kotaria, Archil Guchmanidze, Marina Tediashvili, David Prangishvili

**Affiliations:** 1 G. Eliava Institute of Bacteriophages, Microbiology and Virology, Tbilisi 0160, Georgia; iakusradze@pha.ge (I.K.); t.kokashvili@pha.ge (T.K.); nageli2014@agruni.edu.ge (N.G.); n.janelidze@pha.ge (N.J.); m.tediashvili@pha.ge (M.T.); 2 Richard Lugar Center for Public Health Research, National Center for Disease Control and Public Health, Tbilisi 0198, Georgia; A.kotorashvili@ncdc.ge (A.K.); n.kotaria@ncdc.ge (N.K.); 3 Association “Flora and Fauna”, Batumi 6010, Georgia; guchmanidze@gmail.com; 4 Pasteur Institute, 75015 Paris, France; david.prangishvili@pasteur.fr; 5 Faculty of Medicine, Iv. Javakhishvili Tbilisi State University, Tbilisi 0179, Georgia

**Keywords:** the Black Sea, microbial diversity, phage–host interactions, metagenomics

## Abstract

Viruses have the greatest abundance and highest genetic diversity in marine ecosystems. The interactions between viruses and their hosts is one of the hot spots of marine ecology. Besides their important role in various ecosystems, viruses, especially bacteriophages and their gene pool, are of enormous interest for the development of new gene products with high innovation value. Various studies have been conducted in diverse ecosystems to understand microbial diversity and phage–host interactions; however, the Black Sea, especially the Eastern coastal area, remains among the least studied ecosystems in this regard. This study was aimed at to fill this gap by analyzing microbial diversity and bacteriophage–host interactions in the waters of Eastern Black Sea using a metagenomic approach. To this end, prokaryotic and viral metagenomic DNA from two sampling sites, Poti and Gonio, were sequenced on the Illumina Miseq platform and taxonomic and functional profiles of the metagenomes were obtained using various bioinformatics tools. Our metagenomics analyses allowed us to identify the microbial communities, with *Proteobacteria*, *Cyanobacteria*, *Actinibacteria*, and *Firmicutes* found to be the most dominant bacterial phyla and *Synechococcus* and *Candidatus Pelagibacter* phages found to be the most dominant viral groups in the Black Sea. As minor groups, putative phages specific to human pathogens were identified in the metagenomes. We also characterized interactions between the phages and prokaryotic communities by determining clustered regularly interspaced short palindromic repeats (CRISPR), prophage-like sequences, and integrase/excisionase sequences in the metagenomes, along with identification of putative horizontally transferred genes in the viral contigs. In addition, in the viral contig sequences related to peptidoglycan lytic activity were identified as well. This is the first study on phage and prokaryote diversity and their interactions in the Eastern coastal area of the Black Sea using a metagenomic approach.

## 1. Introduction

Microorganisms play a crucial role in biogeochemical processes and ecosystem functioning of the marine environments. In the last two decades, a special emphasis has been given to the role of viruses in aquatic ecosystems. The interactions between viruses and their hosts is one of the hot spots in marine ecology. Viruses have the greatest abundance and highest genetic diversity in marine ecosystems. Moreover, marine virioplankton influences the composition of microbial populations, causing host death and lysis, thus driving biogeochemical cycles [1,2,3]. An important aspect of phage–host interactions is the lysogeny phenomenon, when temperate bacteriophages integrate in bacterial genomes as prophages, leading to important genetic variations in the host bacteria by transmitting fitness-augmenting genes [4]. Although the integration of temperate phages into the host genome can be beneficial to both the host and phage [5], these prophages may be induced by a wide range of environmental stress factors that lead to lysis of the bacterial cell and phage virion release. Interestingly, prokaryotes have developed a powerful mechanism to fend off from subsequent phage infections. This system, based on a region of DNA called clustered regularly interspaced short palindromic repeats (CRISPR), target DNA or RNA as a way of protecting against viruses and other mobile genetic elements [6]. The CRISPR spacers are often derived from nucleic acid of viruses and plasmids [7], hence keeping records of past viral infections. Therefore, investigation of CRISPR systems provides better insights into virus–host interactions.

Besides their important role in various ecosystems, viruses, especially the bacteriophages and their gene pool, are of enormous interest for the development of new gene products with high innovation value for applications in biotechnology, pharmacy, medicine, and life sciences. Bacteriophages and their enzymes can be used for combating the bacterial infections in various areas such as medicine, veterinary science, and agriculture [8,9]. Moreover, viral DNA and RNA polymerases, ligases, nucleases, recombinases, helicases, and other enzymes have had tremendous success in utility for biotechnological applications and have become classic tools in the molecular biologist’s toolbox [10,11,12].

The Black Sea is the world’s largest anoxic basin. It has an oxygenated surface layer overlying a sulfide-containing (anoxic) deep layer. The sea is connected to the global ocean system through the shallow Bosphorus and Dardanelles straits [13]. The well-stratified water column of the Black Sea; separated oxic, suboxic, and anoxic zones; variations in salinity and temperature; as well as inorganic load creates diverse habitats for microbial assemblages. 

The knowledge on the diversity and role of viruses in microbial communities of the Black Sea is very limited. In particular, little is known about microbial communities of the Eastern Black Sea that are less affected by organic load from the rivers Denube, Dnestr, and Dnepr and are characterized by low chlorophyll *a* content, reduced zooplankton diversity, and low salinity and temperatures [14]. 

Here, we provide the first data on viruses and their host communities as well as virus–host interactions in the Eastern Black Sea using a metagenomic approach. 

## 2. Results

### 2.1. Physico-Chemical Parameters

Both Black Sea sites, Poti and Gonio, were characterized by similar salinity, in a range of 17.2‰ to 17.7‰ (Table 1). The pH was slightly alkaline at both sampling locations, ranging from 7.9 to 8.1. As expected, the water temperature varied considerably by season, with the lowest values in May, being 14 °C and 13 °C for Gonio and Poti, respectively, while in September the water temperature was higher—20 °C at both sites. 

### 2.2. General Characteristics of Prokaryote and Viral Metagenomes

The high-throughput sequencing generated from 2.3 to 6.3 million reads for different prokaryotic and viral DNA samples (Table S1). Following quality and length control, we found that from 1.1 million to 5.6 million reads remained for different samples. The filtered paired-end reads were de novo assembled using CLC Genomics Workbench, resulting in a total of 29,362 contigs with a minimum length of 1000 bp. 

### 2.3. Microbial Community Structure

#### 2.3.1. Prokaryotic Community Structure and Diversity

**Figure 1:**
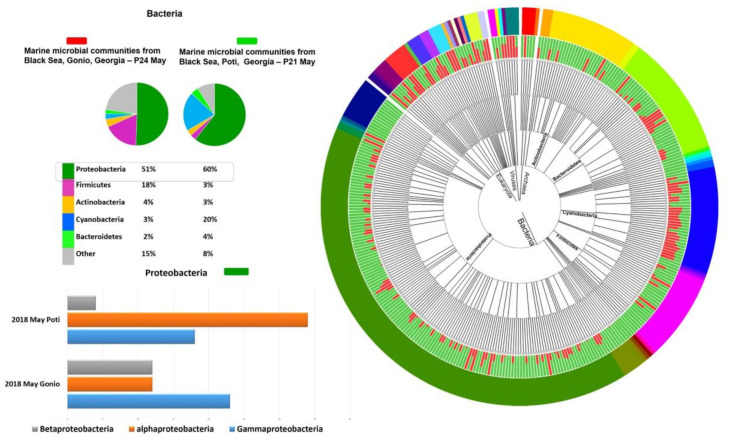
Taxonomic composition of Poti and Gonio metagenomes in May 2018 obtained by alignment of prokaryotic sequence reads to GOTTCHA database.

**Figure 2:**
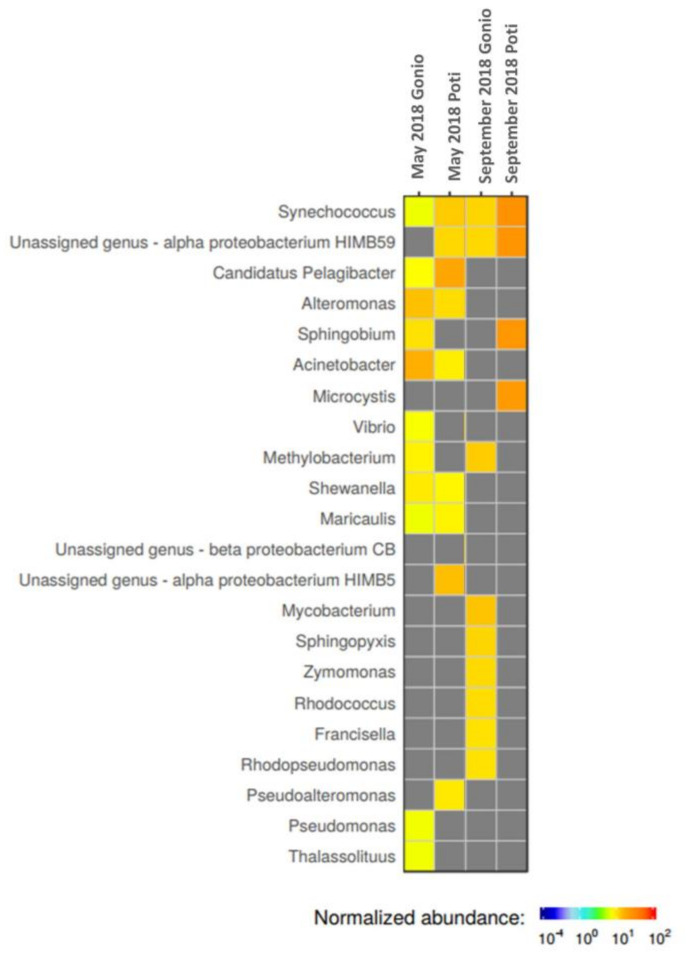
Normalized abundances of microorganisms at the genus level in Poti and Gonio water samples in May and September 2018.

Our studies showed temporal and spatial variations in microbial communities of the Black Sea coastal waters. Notably, in Poti, Proteobacteria at 60% and 38% and Cyanobacteria at 20% and 33% were highly prevalent in May and September samples, respectively (Figure 1). At the class level, Poti samples were dominated by representatives of Cyanobacteria (26%), followed by Alphaproteobacteria (22.5%), Gammaproteobacteria (15%), Betaproteobacteria (7.5%), Actinobacteria (3.5%), and Flavobacteria (2%) (Figure 1). Interestingly, abundance of Cyanobacteria was higher in September compared to the May sample. Seasonal variations were more evident at the genus level. In particular, genera such as *Candidatus Pelagibacter*, alpha proteobacterium HIMB5, *Synechococcus*, *Alteromonas*, *Pseudoalteromonas*, *Acinetobacter*, *Maricaulis*, and *Shewanella* appeared to dominate in May, while *Synechococcus*, alpha proteobacterium HIMB 59, *Sphingobium*, and *Microcystis* were more prevalent in September (Figure 2). 

Gonio waters showed a different microbial composition. Notably, in May, prokaryotic communities, according to the GOTTCHA database, were mainly represented by Proteobacteria (51%) and Firmicutes (18%), while Actinobacteria (48%) and Proteobacteria (28%) prevailed in September (Figure 1). Unlike Poti waters, Cyanobacteria were less prevalent in Gonio, comprising on average only 5% of total metagenomic reads. At the class level in May, the Gonio sample was dominated by Gammaproteobacteria (23%), followed by Bacilli (14%), Alphaproteobacteria (12%), Betaproteobacteria (12%), Mollicutes (7%), Actinobacteria (4%), and Clostridia (4%), while in September, abundance of Actinobacteria increased significantly, comprising 47% of the total microbial community, followed by Alphaproteobacteria (11%), Gammaproteobacteria (9%), Cyanobacteria (7%), and Betaproteobacteria (4%). Prokaryotic taxonomic diversity level showed temporal variation at the genus level as well. In May, the dominant bacterial genera were represented by *Acinetobacter*, *Alteromonas*, *Sphingobium*, *Methylobacterium*, *Synechococcus*, *Shewanella*, *Candidatus Pelagibacter*, *Vibrio*, *Pseudomonas*, *Thalassolituus*, and *Maricaulis*, while in September, *Mycobacterium*, *Methylobacterium*, *Synechococcus*, *Sphingopyxis*, alpha proteobacterium HIMB59, *Zymomonas*, *Rhodococcus*, *Francisella*, and *Rhodopseudomonas* prevailed (Figure 2). 

Alpha diversity that refers to average species diversity in the area was highest in September samples, at 450 and 572 species for Poti and Gonio, respectively, while in May, estimated alpha diversity was relatively lower, at 428 and 409 species for Poti and Gonio, respectively. Such variation can be explained by seasonal differences in physico-chemical parameters of waters. 

#### 2.3.2. Viral Community Structure and Diversity

**Figure 3:**
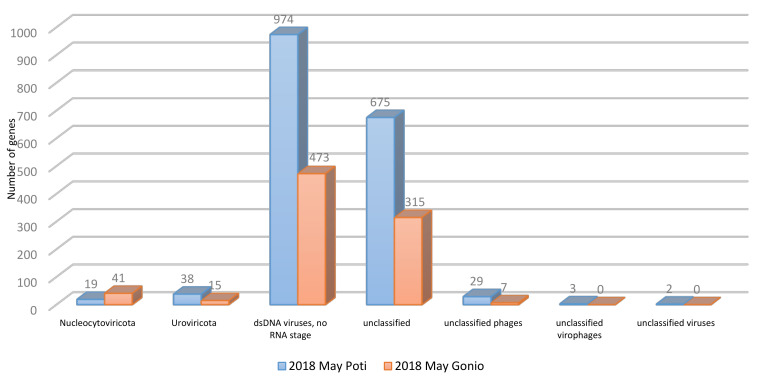
Taxonomic distribution of the Black Sea viral genes.

Classification of the Black Sea viral metagenomic sequences using the GOTTCHA 2 viral database revealed class Caudoviricetes as the dominant group of viruses under the double-stranded DNA (dsDNA) virus phylum in the Georgian coastal areas of the Black Sea (Figure 3). 

**Figure 4:**
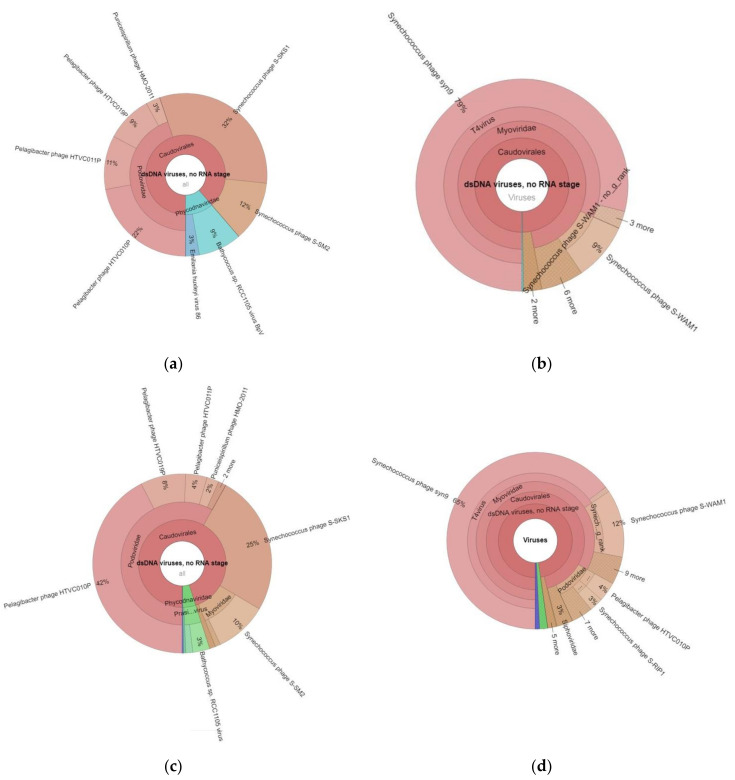
Taxonomic composition of viruses in the Black Sea viral metagenomes obtained by alignment of sequence reads to GOTTCHA 2 viral database. (**a**) Gonio May 2018; (**b**) Gonio September 2018; (**c**) Poti May 2018; (**d**) Poti September 2018.

Representatives of *Caudovirales*, *Myoviridae*, and *Podoviridae* phages appeared as the most dominant viral families in the Black Sea water samples (Figure 4). Sizable portion of genes were attributed to unclassified viruses (Figure 3), indicating that many viruses remain unknown in the Black Sea. Viral communities of Gonio in May were dominated by *Synechococcus* (44%), *Pelagibacter* (42%), and *Puniceispirillum* (3%) phages as well as by viruses of algae, *Bathycoccus* (9%), and *Emiliania huxleyi* (3%), while in September, Gonio waters were almost exclusively dominated by *Synechococcus* phages comprising 94% of total viral community (Figure 4). 

The viral assemblages of the Poti May water sample were dominated by different *Pelagibacter* phages, accounting for 56% of the viral community, followed by *Synechococcus* phages (35%), *Bathycoccus* viruses (3%), and *Puniceispirillum* phages (2%). Similar to Gonio, in September, *Synechoccocus* phages became more prevalent (77%) in Poti waters as well.

As minor groups, putative bacteriophages specific to important human, animal, and plant pathogens were also revealed by our studies (Table S2). In particular, the viral metagenomic sequences were assigned to phages of *Aeromonas*, *Pseudomonas*, *Acinetobacter*, *Vibrio*, *Klebsiella*, *Erwinia*, *Ralstonia*, *Escherichia*, *Salmonella*, and *Campylobacter*. Some of the phage hosts, namely, *Vibrio*, *Aeromonas*, *Pseudomonas*, and *Acinetobacter* are natural inhabitants of the marine environments, while other pathogens can be considered as contaminant microorganisms. The obtained data suggest that the Black Sea waters may harbor phages with potential applications in different fields of medicine, veterinary science, and agriculture. 

### 2.4. Functional Profiles of the Metagenomes

**Figure 5:**
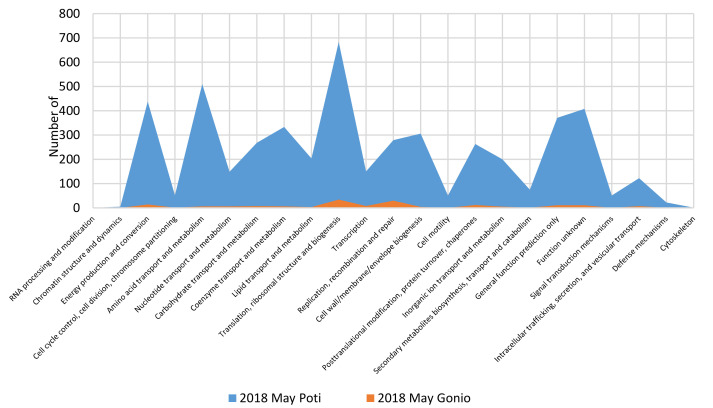
Distribution of the prokaryotic community genes among functional categories of the Pfam pathway.

Functional analysis of prokaryotic contigs against Pfam categories showed similar patterns. The studied metagenomes were enriched with genes critical for the reproduction and survival of microorganisms. Notably, the most functional genes were related to informational and housekeeping functions as well as regulation and metabolism such as “translation, ribosomal structure, and biogenesis”, “amino acid transport and metabolism”, “energy production and conversion”, “carbohydrate transport and metabolism”, “replication, recombination, and repair”, and “posttranslational modification, protein turnover chaperones” (Figure 5). 

**Figure 6:**
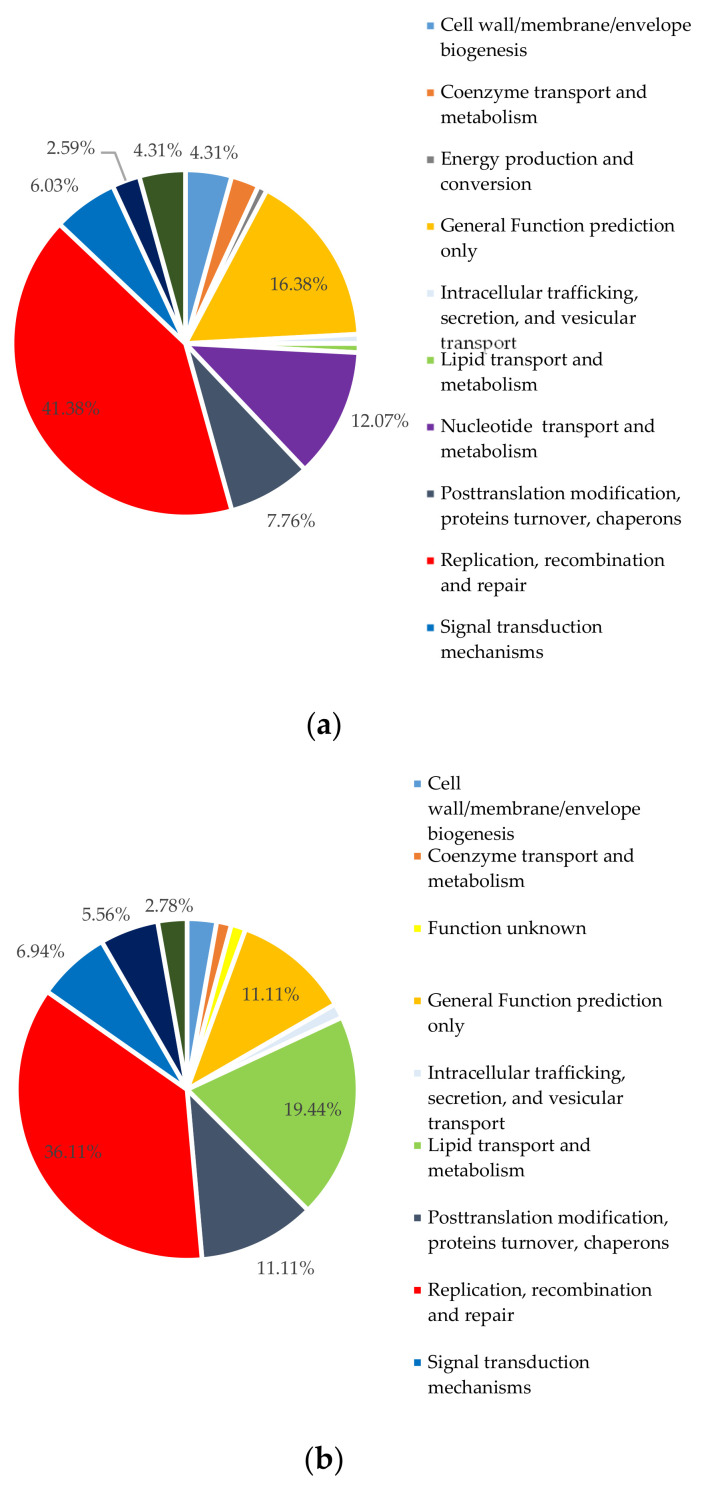
Distribution of viral community genes among functional categories of Pfam pathway. (**a**) Poti May; (**b**) Gonio May.

Functional analysis of all viral metagenomic sequences against Pfam categories also displayed similar trends. The most frequent genes in the viral predicted genes were related to processes vital to phage production such as “replication, recombination, and repair”. Other major functions were “nucleotide transport and metabolism” and “posttranslational modification, protein turnover chaperones” categories (Figure 6). 

Remarkably, the majority of annotated genes did not fall into Pfam categories, indicating that the Black Sea viruses have genetic makeups very different from those available in the public databases.

### 2.5. Phage–Host Interactions

#### 2.5.1. Prophage Identification

Identification of prophages in the prokaryotic contigs is essential for better characterization of phage–host interactions in the natural ecosystems. Using a PHASTER tool, a total of 13 prophage-like sequences, 8 from Poti September and 5 from Gonio September, ranging from 6.1 to 42.3 kbp, were retrieved from prokaryotic metagenomes (Table S3). Absence of putative prophage sequences in May samples can be explained by low numbers of contigs retrieved from May prokaryotic metagenomes compared to September samples (Table S1). The majority of putative prophage sequences were assigned to *Synechococcus* phages, some of which belonged to the dominant phage groups such as Syn9, S-SKS1, S-SM2, and S_WAM1 found in the Black Sea viral metagenomes. 

#### 2.5.2. Integrase/Excisionase Identification

Besides performing identification of prophage-like sequences, we also studied the presence of integrase and excisionase genes in the prokaryotic contigs. It should be noted that establishment of lysogeny requires bacteriophage expression of integrase, while excision requires an additional phage-encoded protein called excisionase. Therefore, these two proteins are considered markers of lysogenic infection [15].

**Figure 7:**
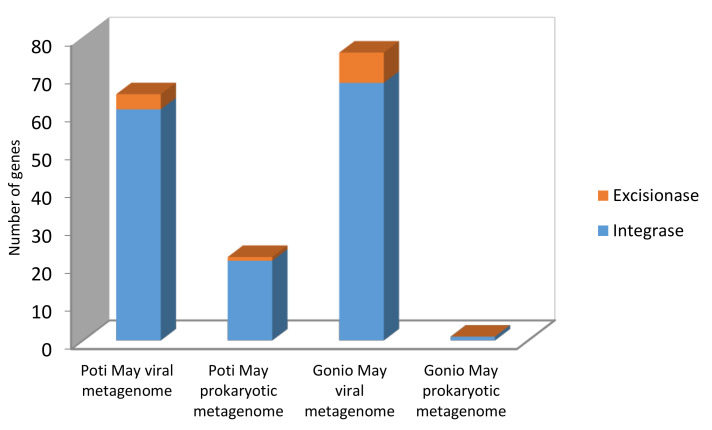
Occurrence of integrase/excisionase genes in the Black Sea prokaryotic and viral metagenomes.

Using the IMG/M tool, a total of 164 proteins were annotated as integrases or excisionases in the Black Sea prokaryotic and viral metagenomes. The majority of these genes, 132 integrases and 12 excisionases, were found among the viral metagenomic samples, and the rest, 21 integrase and 1 excisionase genes, were identified in prokaryotic metagenomes (Figure 7). The bacteria harboring the marker genes of lysogenic infections were different families of marine microbes such as *Alteromonadaceae*, *Litoricolaceae*, *Rhodobacteraceae*, *Oceanospirillaceae*, *Flavobacteriaceae*, *Sphingobacteriaceae*, and *Pseudoalteromonadaceae*. Our studies suggest that the microbial populations of the Black Sea are enriched with lysogenic infections.

#### 2.5.3. CRISPR Identification

CRISPRs constitute a memory of past viral invasions, therefore representing valuable records of virus–host interactions. Using the IMG/M tool, we recognized 12 CRISPR arrays from all bacterial and viral contigs, with an average repeat length of 27 bp and an average spacer length of 39 bp. The regions were linked to the dominant bacterial phyla in the Black Sea, such as *Flavobacteriaceae*, *Pelagibacteriaceae*, *Microcystaceae*, and unclassified Sphingomonadales (Table S4). Interestingly, three regions were attributed to *Caudovirales* phages and unclassified marine viruses (Table S4). Five regions could not be assigned to any known microbial sequences in the National Center for Biotechnology Information (NCBI) database.

#### 2.5.4. Putative Horizontally Transferred Genes

On the basis of best hits to genes from bacteria, we analyzed the presence of putative horizontally transferred genes in the contigs of dominant phage groups (*Synechococcus* phages syn9, S-SM2, S-SKS1; Pelagibacter phages HTVC010P, HTVC011P, HTVC019P) and selected phages specific to pathogenic bacteria using the IMG/M online tool (Table S5). The best hits with bacterial genomes were found in *Synechococcus* phages syn9 and S-SM2. In total, 11 and 15 genes with hypothetical or known protein functions in Syn9 and S-SM2 were identified, respectively. Among these proteins, the most remarkable were enzymes associated with restriction–modification system, such as modification methylase BamHII, a methyltransferase family protein, which provides protection against restriction endonucleases, as well as proteins responsible for DNA replication and transcription and putative plasmid mobilization protein, responsible for mobilization of genetic elements. In pelagibacter phages, no putative horizontally transferred genes were found using the aforementioned bioinformatics tool. 

In genomes of phages specific to pathogens, the most notable best hits were related to endonucleases, carbohydrate transport, assembly of transmembrane proteins, enzymes involved in sensing and responding to environmental stimuli, synthesis of amino acids, efflux transporters conferring multidrug resistance to bacteria, RNA and DNA synthesis, survival under extreme conditions, and mobilization of genetic elements. Among the selected phages, the highest number of putative horizontally transferred genes was found among *Pseudomonas* phages (Table S5).

### 2.6. Identification of Peptidoglycan Hydrolase-Encoding Genes

As the marine viruses harbor an enormous pool of genes with biotechnological interest, we tried to identify peptidoglycan hydrolase-encoding genes in the Black Sea DNA metagenomes. Virion-associated peptidoglycan hydrolases (VAPGHs), analogous to endolysins, are phage-encoded lytic enzymes that specifically degrade peptidoglycan. VAPGHs can be considered as antimicrobial agents for eradication of pathogens (both in medicine and in industry or biotechnological settings) [16]. 

In our studies, the clusters of orthologous groups of proteins (COG) pathway recognized eight sequences related to peptidoglycan lytic activity among the assembled viral contigs: four were found in *Pelagibacter* phages belonging to Myoviruses, one was linked to Siphovirus uncultured Mediterranean phage uvMED, and three to unclassified marine phages (Table S6). 

Since peptidoglycan hydrolases of bacterial origin can also be used as antimicrobials, we also checked prokaryotic contigs for the presence of genes encoding these enzymes. Lower numbers of peptidoglycan hydrolase genes were found among prokaryotic metagenomes: two genes were linked to *Candidatus Pelagibacter*, one was linked to *Flavobacteria*, and one gene could not be assigned to any known sequence in the database. Uncharacterized N-terminal domain of peptidoglycan hydrolase, CwlO, was the major type of peptidoglycan lytic activity associated with sequences found in marine phages in the Black Sea. 

## 3. Discussion

### 3.1. Microbial Diversity

In this work, we used viral and bacterial metagenome sequencing to characterize the microbial communities and the virus–host interactions in the Black Sea. The Black Sea belongs to the brackish water ecosystems with salinity much lower than that of the Mediterranean Sea to which the Black Sea is connected through the narrow Bosporus and Dardanelles straits [14]. Despite its low salinity, according to our research, the Black Sea is dominated by microbial communities widely distributed in the marine habitats. Namely, a quite sizable portion of reads were assigned to *Candidatus Pelagibacter*, alpha proteobacterium HIMB59, and alpha proteobacterium HIMB5 (Figure 2), pointing towards the high abundance of SAR 11 clade Alphaproteobacteria in the Black Sea. SAR11 is a group of small, carbon-oxidizing bacteria found in the oceans, being the most abundant heterotrophic bacteria, which play a major role in carbon cycling [17]. Seasonal cycles of SAR11 clade and *Candidatus Pelagibacter* were described by different authors [18,19]. We observed high abundance of *Candidatus Pelagibacter* in May compared to the September sample. Such changes in abundance may depend on different environmental factors, such as interactions with phytoplankton, availability of dissolved organic matter (DOM), grazing by eukaryotes, or predation by viruses [20,21]. Besides the SAR11 clade, we also found halophylic Alphaproteobacterium *Maricaulis* belonging to the Rhodobacteriales order, the bacteria described to be widely distributed in marine habitats and considered to be rapid surface colonizers in ocean waters [22]. Another representative of Alphaproteobacteria, *Methylobacterium*, which dominated the Gonio prokaryotic communities, is ubiquitous in nature and can be found in almost any freshwater environment where dissolved oxygen exists [23], as well as in marine ecosystems [24]. Similar to microbial assemblages of oceans [25] and various marine habitats [26,27], Gammaproteobacteria also dominated the Black Sea microbial communities. Typical marine bacteria, such as *Alteromonas*, *Shewanella*, and *Pseudoalteromonas*, along with the clinically important Gammaproteobacteria, *Acinetobacter*, *Vibrio*, and *Pseudomonas*, were the major genera in the Black Sea coastal waters (Figure 2). *Alteromonas* and *Pseudoalteromonas* species are globally distributed copiotrophic bacteria inhabiting nutrient rich marine ecosystems [28,29]. These species thrive in the habitats with oil spills or in decaying phytoplankton blooms [29], often being observed in the Black Sea as well [30]. *Acinetobacter* and *Pseudomonas* are ubiquitous bacteria in the environment, including the marine ecosystems [31], and are also known to cause drug-resistant-related community and hospital-acquired infections [32,33]. We assume that the high abundance of *Acinetobacter* and *Pseudomonas* in the Black Sea coastal waters can be considered as a risk factor for increased colonization and incidence of *Acinetobacter* and *Pseudomonas* infections in the community. 

Interestingly, Gonio coastal waters harbor abundant groups of microbes not found in high prevalence in Poti area. We have revealed the presence of *Mycobacteria* (Actinobacteria), *Sphingopyxis* (Alphaproteobacteria), *Zymomonas* (Alphaproteobacteria), *Rhodococcus* (Actinobacteria), *Francisella* (Gammaproteobacteria), and *Rhodopseudomonas* (Alphaproteobacteria), representing the dominant groups in the Gonio September sample. Actinobacteria appeared to be the most dominant class in Gonio in September. These bacteria have been primarily thought of as soil bacteria. However, several studies, using different approaches, have shown now that Actinobacteria are very common and abundant members of freshwater as well as marine communities [34,35]. Furthermore, more recently it was shown [35] that marine actinobacteria, notably members of genus *Streptomyces*, may serve as a source for potential new drugs against multidrug-resistant bacteria. We have found representatives of the genus *Streptomyces* at both sampling sites, indicating that the Black Sea may serve as a source for Actinobacteria producing biologically active compounds of therapeutic potential. Besides its production of antimicrobials, *Streptomyces* is an important organism in carbon recycling that has a crucial role in the environment since it carries out a broad range of metabolic processes such as degradation of insoluble biological material including lignocellulose and chitin [36]. The most dominant representatives of Actinobacteria in our metagenomes were *Mycobacteria*, which dominated the Gonio waters in September. The presence of these bacteria in marine waters is not surprising, as *Mycobacteria* were reported to cause the infections in fresh and marine water fish [37]. In the Black Sea water samples, we also found bacteria capable of degrading crude oil, such as *Sphingobium, Sphingopyxis*, and *Thalassolituus*. Prevalence of these bacteria can be explained by contamination of the Black Sea waters with oil from the oil terminals, cruise ships, tankers, and tourist boats. Cultivation and characterization of these bacteria from the Black Sea water samples can be considered for future studies with remediation purposes. 

*Synechococcus* are ubiquitous and cosmopolitan cyanobacteria that play important roles in global productivity and biogeochemical cycles [38,39]. We found a high abundance of *Synechococcus* (Figure 2) in the Black Sea waters that was in agreement with previous studies in other marine waters and estuaries worldwide, showing dominance of these cyanobacteria particularly in coastal areas where waters are enriched with nitrate [40]. Temperature was also reported as one of the most important parameters determining the abundance of *Synechococcus* [38,41], which is consistent with our data showing a higher proportion of *Synechococcus* reads in September, when the water temperature was higher (Table 1) in comparison to the May sample.

Similar to marine and other aquatic environments [42,43], *Caudovirales*, tailed bacteriophages, were found to dominate the Black Sea viral communities. We observed predominance of phages specific to highly prevalent bacterial groups, *Candidatus Pelagibacter* and *Synechococcus*. Interestingly, September samples at both locations were almost exclusively dominated by *Synechococcus* phage Syn9, while viral communities in May were represented by *Synechococcus* phages S-SKS1 and S-SM2 and *Pelagibacter* phages HTVC010P, HTVC011P, and HTVC019P. Syn9 is a representative of an abundant group of T4-like cyanophages in oceans, resembling the T4 coliphage archetype, both in virion morphology and core gene content [44,45]. Pelagiphages HTVC011P, HTVC019P, and HTVC-010P morphologically belong to the short-tailed Podoviridae family that is widely distributed in marine ecosystems. HTVC010P was found in high numbers in ocean surface waters and has been proposed as one of the most abundant virus subfamilies in the biosphere [46]. Similar to Syn9, *Synechococcus* phages S-SKS1 and S-SM2 are also myoviruses widely distributed in various marine environments [47,48]. 

Despite a high prevalence of Actinobacteria, especially in the Gonio September sample, we found a small number of phages specific to Actinobacteria in the water samples (Table S2). We presumed a higher abundance of Actinobacteria phages in the Black Sea but it is likely these were mainly unknown phages, and consequently their sequences were not deposited in the databases. 

In the studied viral metagenomes, we found sequences related to the phages of pathogenic bacteria. Presence of phages specific to *Vibrio*, *Aeromonas*, *Pseudomonas, Acinetobacter*, and of other autochthonous bacteria is not surprising as the phages follow distribution of their host. Occurrence of vibrio phages in the Black Sea was reported previously [49] as well. Currently, the G. Eliava Institute of Bacteriophages, Microbiology and Virology keeps in the collection over 20 vibrio phages specific to *Vibrio cholerae*, *Vibrio parahaemolyticus*, and other clinically important vibrio species (Dr. T. Kokashvili, G. Eliava Institute of Bacteriophages Microbiology and Virology, Tbilisi, Georgia, personal communication). Moreover, as the Georgian coastal waters of the Black Sea are under increased anthropogenic impact, especially during the warm season, values of microbial contamination are elevated [49], and thus the presence of phages specific to *Escherichia coli*, *Salmonella*, *Klebsiella*, and other human pathogens can be expected. It should be also noted that the Black Sea water samples are often used by researchers for isolation of bacteriophages against different human, fish, and plant pathogens such as *Escherichia coli*, *Pseudomonas aeruginosa*, *Pseudomonas fluorescens*, *Aeromonas hydrophila*, *Erwinia carotovora*, and others (Dr. M. Tediashvili and Dr. N. Janelidze, G. Eliava Institute of Bacteriophages Microbiology and Virology, Tbilisi, Georgia, personal communication). These data suggest that the Black Sea serves as a good source for isolation of phages against various infectious agents.

### 3.2. Phage–Host Interactions

In this study, we tried to characterize some aspects of the phage–host interactions by determining the prophage, CRISPR integrase, and excisionase sequences in the Black Sea metagenomes. 

The high prevalence of prophages in marine systems has been recognized for a decade. Researchers found that about half of marine bacterial genomes contain prophage-like elements [50]. In the Black Sea prokaryotic metagenomes, we found prophage-like sequences that were mainly related to *Synechococcus* phages. This was not surprising, as it has been demonstrated that lysogeny is a common phenomenon in natural populations of marine *Synechococcus* [51,52]. 

Although we could not find prophage sequences related to *Candidatus Pelagibacter* phages in the assembled contigs, the presence of pelagiphages HTVC011P and HTVC019P that show a temperate nature [53] gives us the clue that the lysogeny is the widespread type of phage–host interaction in the Black Sea. Our assumption is further strengthened by the high abundance of integrase/excisionase genes in the assembled contigs. Our research suggests that dominating *Synechococcus* as well as *Pelagibacter* phages may influence their host populations by a variety of mechanisms, including mortality, genetic transduction, and prophage-induced viral immunity in the Black Sea. 

Identification of CRISPRs in the bacterial contigs of the Black Sea metagenomes indicates the prevalence of previous infections among the prokaryotic community. We found CRISPR arrays mainly in dominant bacterial groups, including *Pelagibacteriaceae*, bacteria that rarely use CRISPR/Cas or restriction–modification systems for phage defense [17,54]. Interestingly, we identified CRISPR sequences among viral contigs as well (Table S4). Previous works [55,56] have also found CRISPRs in phage genomes. One study [55] showed that the phage-encoded CRISPR/Cas system is used to counteract a phage inhibitory chromosomal island of the bacterial host. It has also been suggested that the compact CRISPR/Cas system identified in huge bacteriophages, which is active in human and plant cells, might be successfully used for genome editing [56]. The presence of CRISPRs in marine viral contigs shows that marine bacteriophages may provide valuable CRISPR/Cas system enzymes for the genome editing toolbox.

Phages also contribute to the genetic diversification of prokaryotic communities through transduction, a form of phage-mediated horizontal gene transfer (HGT). Transduction has been intensively studied in various natural environments including marine waters [57]. Between 1.6 and 32.6% of genes in prokaryote genomes are estimated to have been acquired through horizontal gene transfer [58]. Interestingly, research studying the susceptibility of marine microbes to infection by phages isolated from soil, marine sediments, and fresh water has demonstrated that phages propagate and move between major biomes, mediating the transfer of DNA between microbes from very different ecosystems [59]. According to our studies, horizontal gene transfer appears to play an important role in genetic recombination between microorganisms in the Black Sea. The most notable putative horizontally transferred genes found in bacteriophage contigs and reference bacterial genomes were related to RNA and DNA synthesis, response to environmental stimuli, antibiotic resistance, and mobilome, once again confirming the importance of phages in augmenting the fitness of host bacteria under different environmental conditions. In our study, the putative horizontally transferred genes were found in *Synechococcus* phages as well as in phages of pathogenic bacteria, indicating the importance of complete characterization of phage biological and genetic properties prior to their therapeutic applications.

### 3.3. Phage-Derived Peptidoglycan Hydrolases

Bacteriophages produce a variety of enzymes capable of degrading the bacterial cell wall and therefore are considered for biotechnological applications [60]. We tried to identify genes encoding peptidoglycan hydrolases in assembled prokaryotic and viral contigs. Uncharacterized N-terminal domain of peptidoglycan hydrolase CwlO was the major type of peptidoglycan lytic activity-associated sequences found in marine phages in the Black Sea. CwlO belongs to D,L-endopeptidases, and the presence of this motif in proteins was reported to be associated with peptidoglycan lytic activity [61]. The sequences linked to virion-associated muralytic activities were found in genome sequences of various prophages and phages infecting Gram-positive as well as Gram-negative bacteria [60]. In our study, we found sequences related to peptidoglycan hydrolase genes in widely distributed phages of *Pelagibacter* and some other marine viruses. To date, there is very limited information about virion-associated muralytic activities in marine viruses. Further research is needed to reveal and characterize the biotechnological potential of antimicrobial enzymes encoded by marine viruses. 

## 4. Materials and Methods

### 4.1. Sample Collection

Surface seawater samples (Table 1) were collected in May and September 2018 from two sites, Poti and Gonio, at the eastern coastal area the Black Sea. Measurements of hydrochemical parameters, such as temperature, salinity, and pH, were completed on-site using a portable multilog meter (YSI 556 MPS, Yellow Springs Instruments, Yellow Springs, CO, USA). 

### 4.2. Sample Processing and DNA Extraction

Twenty liters of each water sample were concentrated by filtration through 0.22 μm polyvinylidene difluoride (PVDF) filters (Millipore, Burlington, MA, USA). The remaining filtrate was used for concentration of viruses (see below). DNA was extracted from the filters using a Promega Wizard Genomic DNA Purification Kit (Promega, Madison, WI, USA), according to the manufacturer’s instructions. 

Viruses from seawater were concentrated using iron-based flocculation and large-pore-size filtration technique, as previously described [62], with some modifications. Briefly, 5 L of seawater was mixed with 0.25 mL of 10 g/L Fe stock (FeCl_3_·6H_2_O) solution and incubated at room temperature for 1 h. Fe-virus precipitate was collected onto a 90 mm diameter 1.0 µm polycarbonate membrane filter. For effective virus resuspension from polycarbonate membrane filters, 5 mL of resuspension buffer containing 0.125 M Tris base, 0.1 M EDTA, 0.1 M MgCl_2_·6H_2_O, and 0.2 M oxalic acid was added to 5 L seawater precipitate, which was incubated overnight with shaking at 4 °C in the dark. DNA was extracted from the suspension using a Promega Wizard Genomic DNA Purification Kit (Promega, Madison, WI, USA), according to the manufacturer’s instructions. 

DNA extracts of prokaryotic and viral samples were sequenced at the Lugar’s Center of the National Center of Disease Control and Public Health, Tbilisi, Georgia, using the Illumina Miseq Platform (Illumina, San Diego, CA, USA). The DNA was sheared using the Covaris M220-focused ultrasonicator (Covaris, Inc., Woburn, MA, USA) and the quality control performed on the BioAnalyzer 2100 (Agilent Technologies, Inc. Santa Clara, CA, USA). Library preps were quantified using the Qubit 2.0 Fluorometer (Life Technologies, Carlsbad, CA, USA). Fragment size analysis was performed on a BioAnalyzer 2100 (Agilent Technologies, Inc., Santa Clara, CA, USA).

### 4.3. Bioinformatics Analysis

The obtained sequence reads were checked for quality by Quality Control Software and trimmed using CLC Genome Workbench (CLC Bio, AArhus, Denmark). PhiX174 phage reads (NC_001422.1), used as an internal control in Illumina sequencing, were removed by mapping to reference.

For functional analysis, the filtered reads were de novo assembled using CLC Genomics Workbench. Contigs less than 1000 bp were removed, and the remaining sequences were submitted to The Integrated Microbial Genomes and Microbiomes system v.5.0 (IMG/M) [63] for identification of protein-coding genes and functional annotations using Clusters of Orthologous Groups of proteins (COG), Protein families (Pfam), and HMMER () pathways. Prophage and CRISPR sequences were identified using IMG/M software (Version No. 5.0), and the results of prophage sequence analysis were confirmed by online PHASTER tool (PHAge Search Tool Enhanced Release) [64].

Taxonomic analysis was performed using the EDGE Bioinformatics online tool (version EDGE-UI v2.4.0) [65]. For assigning taxonomic labels to metagenomic prokaryotic and viral DNA sequences, Kraken [66], Genomic Origins Through Taxonomic CHAllenge (GOTTCHA) (bacterial species database), and GOTTCHA 2 (viral species database) were used. Metagenomic datasets are available in the National Center for Biotechnology Information (NCBI) under accession numbers SAMN16453361 (Gonio P-24), SAMN16453362 (Gonio BS29), SAMN16453363 (Poti P-21), SAMN16453364 (Poti BS26), SAMN16453365 (Gonio P34), SAMN16453366 (Poti P31), and SAMN16453367 (Poti BS36).

Alpha-diversity of the datasets were calculated using MG-RAST (metagenomics rapid annotation using subsystem technology) [67].

## Figures and Tables

**Table 1:** Location and physico-chemical parameters of the Black Sea sampling sites.

Sampling Sites and Time	T °C	pH	Salinity %	Location
Gonio, May 2018	14	8.0	17.2	41°36′29.9″ N 41°32′41.8″ E
Gonio, September 2018	20	8.1	17.5	41°36′29.9″ N 41°32′41.8″ E
Poti, May 2018	13	8.1	17.7	42°08′59.5″ N 41°38′16.1″ E
Poti, September 2018	20	7.9	17.2	42°08′59.5″ N 41°38′16.1″ E

## Supplementary Materials

The following are available online at https://www.mdpi.com/1660-3397/18/11/558/s1, Table S1: General characteristics of the Black Sea metagenomes; Table S2. List of putative phages identified in the Black Sea viral metagenomes; Table S3. Putative prophages in the Black Sea prokaryotic contigs; Table S4. CRISPR arrays in the Black Sea prokaryotic and viral metagenomes; Table S5. Genes in phages with Best Hits to Genes from Bacteria/Putative Horizontally Transferred Genes in some of the viral contigs; Table S6. Occurrence of peptidoglycan hydrolase genes in the Black Sea metagenomes.

## References

[1] Suttle C.A. (2007). Marine viruses—Major players in the global ecosystem. Nat. Rev. Genet..

[2] Rohwer F., Prangishvili D., Lindell D. (2009). Roles of viruses in the environment. Environ. Microbiol..

[3] Middelboe M., Brussaard C.P.D. (2017). Marine Viruses: Key Players in Marine Ecosystems. Viruses.

[4] Pleška M., Lang M., Refardt D., Levin B.R., Guet C.C. (2018). Phage–host population dynamics promotes prophage acquisition in bacteria with innate immunity. Nat. Ecol. Evol..

[5] Yosef I., Manor M., Kiro R., Qimron U. (2015). Temperate and lytic bacteriophages programmed to sensitize and kill antibiotic-resistant bacteria. Proc. Natl. Acad. Sci. USA.

[6] Barrangou R., Fremaux C., Deveau H., Richards M., Boyaval P., Moineau S., Romero D.A., Horvath P. (2007). CRISPR Provides Acquired Resistance Against Viruses in Prokaryotes. Science.

[7] Mojica F.J.M., Soria E. (2005). Intervening Sequences of Regularly Spaced Prokaryotic Repeats Derive from Foreign Genetic Elements. J. Mol. Evol..

[8] Chanishvili N. (2016). Bacteriophages as Therapeutic and Prophylactic Means: Summary of the Soviet and Post Soviet Experiences. Curr. Drug Deliv..

[9] Harada L.K., Silva E.C., Campos W.F., Del Fiol F.S., Vila M., Dąbrowska K., Krylov V.N., Balcão V.M. (2018). Biotechnological applications of bacteriophages: State of the art. Microbiol. Res..

[10] Blondal T., Hjorleifsdottir S.H., Fridjonsson O.F., Aevarsson A., Skirnisdottir S., Hermannsdottir A.G., Hreggvidsson G.O., Smith A.V., Kristjansson J.K. (2003). Discovery and characterization of a thermostable bacteriophage RNA ligase homologous to T4 RNA ligase 1. Nucleic Acids Res..

[11] Makeyev E.V., Grimes J.M. (2004). RNA-dependent RNA polymerases of dsRNA bacteriophages. Virus Res..

[12] Born E.V.D., Omelchenko M.V., Bekkelund A., Leihne V., Koonin E.V., Dolja V.V., Falnes P.Ø. (2008). Viral AlkB proteins repair RNA damage by oxidative demethylation. Nucleic Acids Res..

[13] Stewart K., Kassakian S.Z., Krynytzky M., DiJulio D., Murray J.W. (2006). Oxic, suboxic, and anoxic conditions in the Black Sea. The Black Sea Flood Question: Changes in Coastline, Climate, and Human Settlement.

[14] Zaitsev Y.P., Alexandrov B.G., Berlinsky N.A. The Black Sea: An Oxygen-Poor Sea. Europe’s Biodiversity-Biogeographical Regions and Seas. http://www.vliz.be/en/imis?module=ref&refid=26842&printversion=1&dropIMIStitle=1.

[15] Knowles B., Silveira C.B., Bailey B.A., Barott K., Cantu V.A., Cobián-Güemes A.G., Coutinho F.H., Dinsdale E.A., Felts B., Furby K.A. (2016). Erratum: Corrigendum: Lytic to temperate switching of viral communities. Nat. Cell Biol..

[16] Rodríguez-Rubio L., Martínez B., Donovan D.M., Rodríguez A., García P. (2012). Bacteriophage virion-associated peptidoglycan hydrolases: Potential new enzybiotics. Crit. Rev. Microbiol..

[17] Giovannoni S.J. (2017). SAR11 Bacteria: The Most Abundant Plankton in the Oceans. Annu. Rev. Mar. Sci..

[18] Carlson C., Morris R., Parsons R., Treusch A.H., Giovannoni S.J., Vergin K. (2008). Seasonal dynamics of SAR11 populations in the euphotic and mesopelagic zones of the northwestern Sargasso Sea. ISME J..

[19] Bowman J.S., Amaral-Zettler L., Rich J.J., Luria C.M., Ducklow H.W. (2017). Bacterial community segmentation facilitates the prediction of ecosystem function along the coast of the western Antarctic Peninsula. ISME J..

[20] Delmont T.O., Hammar K.M., Ducklow H.W., Yager P.L., Post A.F. (2014). Phaeocystis antarctica blooms strongly influence bacterial community structures in the Amundsen Sea polynya. Front. Microbiol..

[21] Kudo T., Kobiyama A., Rashid J., Reza S., Yamada Y., Ikeda Y., Ikeda D., Mizusawa N., Ikeo K., Sato S. (2018). Seasonal changes in the abundance of bacterial genes related to dimethylsulfoniopropionate catabolism in seawater from Ofunato Bay revealed by metagenomic analysis. Gene.

[22] Dang H., Li T., Chen M., Huang G. (2007). Cross-Ocean Distribution of Rhodobacterales Bacteria as Primary Surface Colonizers in Temperate Coastal Marine Waters. Appl. Environ. Microbiol..

[23] Gallego V., García M.T., Ventosa A. (2005). Methylobacterium hispanicum sp. nov. and Methylobacterium aquaticum sp. nov., isolated from drinking water. Int. J. Syst. Evol. Microbiol..

[24] Lee J.-W., Nam J.-H., Kim Y.-H., Lee K.-H., Lee D.-H. (2008). Bacterial communities in the initial stage of marine biofilm formation on artificial surfaces. J. Microbiol..

[25] Biers E.J., Sun S., Howard E.C. (2009). Prokaryotic Genomes and Diversity in Surface Ocean Waters: Interrogating the Global Ocean Sampling Metagenome. Appl. Environ. Microbiol..

[26] Bobrova O., Kristoffersen J.B., Oulas A., Ivanytsia V. (2016). Metagenomic 16s rRNA investigation of microbial communities in the Black Sea estuaries in South-West of Ukraine. Acta Biochim. Pol..

[27] Tseng C.-H., Chiang P.-W., Lai H.-C., Shiah F.-K., Hsu T.-C., Chen Y.-L., Wen L.-S., Tseng C.-M., Shieh W.Y., Saeed I. (2015). Prokaryotic assemblages and metagenomes in pelagic zones of the South China Sea. BMC Genom..

[28] Math R.K., Jin H.M., Kim J.M., Hahn Y., Park W., Madsen E.L., Jeon C.O. (2012). Comparative Genomics Reveals Adaptation by Alteromonas sp. SN2 to Marine Tidal-Flat Conditions: Cold Tolerance and Aromatic Hydrocarbon Metabolism. PLoS ONE.

[29] Duhaime M.B., Wichels A., Sullivan M.B. (2016). Six Pseudoalteromonas Strains Isolated from Surface Waters of Kabeltonne, Offshore Helgoland, North Sea. Genome Announc..

[30] Kubryakov A.A., Mikaelyan A.S., Stanichny S.V. (2019). Summer and winter coccolithophore blooms in the Black Sea and their impact on production of dissolved organic matter from Bio-Argo data. J. Mar. Syst..

[31] Yoon J.-H., Kim I.-G., Oh T.-K. (2007). Acinetobacter marinus sp. nov. and Acinetobacter seohaensis sp. nov., isolated from sea water of the Yellow Sea in Korea. J. Microbiol. Biotechnol..

[32] Doughari H.J., Ndakidemi P.A., Human I.S., Benade S. (2011). The Ecology, Biology and Pathogenesis of Acinetobacter spp.: An Overview. Microbes Environ..

[33] Nair A.V., Joseph N., Krishna K., Sneha K.G., Tom N., Jangid K., Nair S. (2015). A comparative study of coastal and clinical isolates of Pseudomonas aeruginosa. Braz. J. Microbiol..

[34] Allgaier M., Grossart H.-P. (2006). Diversity and Seasonal Dynamics of Actinobacteria Populations in Four Lakes in Northeastern Germany. Appl. Environ. Microbiol..

[35] Ibrahimi M., Korichi W., Hafidi M., Lemee L., Ouhdouch Y., Loqman S. (2020). Marine Actinobacteria: Screening for Predation Leads to the Discovery of Potential New Drugs against Multidrug-Resistant Bacteria. Antibiotics.

[36] Bentley S.D., Chater K.F., Cerdeño-Tárraga A.-M., Challis G.L., Thomson N.R., James K.D., Harris D.E., Quail M., Kieser H.M., Harper D.P. (2002). Complete genome sequence of the model actinomycete Streptomyces coelicolor A3(2). Nat. Cell Biol..

[37] Hashish E., Merwad A., Elgaml S., Amer A., Kamal H., Elsadek A., Marei A., Sitohy M. (2018). Mycobacterium marinum infection in fish and man: Epidemiology, pathophysiology and management; a review. Vet. Q..

[38] Flombaum P., Gallegos J.L., Gordillo R.A., Rincón J., Zabala L.L., Jiao N., Karl D.M., Li W.K.W., Lomas M.W., Veneziano D. (2013). Present and future global distributions of the marine Cyanobacteria Prochlorococcus and Synechococcus. Proc. Natl. Acad. Sci. USA.

[39] Sohm J., Ahlgren N., Thomson Z.J., Williams C., Moffett J.W., Saito M., Webb E.A., Rocap G. (2015). Co-occurring Synechococcus ecotypes occupy four major oceanic regimes defined by temperature, macronutrients and iron. ISME J..

[40] Affe H.M.D.J., Rigonato J., Nunes J.M.D.C., Menezes M. (2018). Metagenomic Analysis of Cyanobacteria in an Oligotrophic Tropical Estuary, South Atlantic. Front. Microbiol..

[41] Chiang K.-P., Kuo M.-C., Chang J., Wang R.-H., Gong G.-C. (2002). Spatial and temporal variation of the Synechococcus population in the East China Sea and its contribution to phytoplankton biomass. Cont. Shelf Res..

[42] Weinbauer M.G. (2004). Ecology of prokaryotic viruses. FEMS Microbiol. Rev..

[43] Winter C., Garcia J.A.L., Weinbauer M.G., Dubow M.S., Herndl G.J. (2014). Comparison of Deep-Water Viromes from the Atlantic Ocean and the Mediterranean Sea. PLoS ONE.

[44] Mann N.H., Clokie M.R.J., Millard A., Cook A., Wilson W.H., Wheatley P.J., Letarov A., Krisch H.M. (2005). The Genome of S-PM2, a “Photosynthetic” T4-Type Bacteriophage That Infects Marine Synechococcus Strains. J. Bacteriol..

[45] Weigele P.R., Pope W.H., Pedulla M.L., Houtz J.M., Smith A.L., Conway J.F., King J., Hatfull G.F., Lawrence J.G., Hendrix R.W. (2007). Genomic and structural analysis of Syn9, a cyanophage infecting marine Prochlorococcus and Synechococcus. Environ. Microbiol..

[46] Zhao Y., Temperton B., Thrash J.C., Schwalbach M.S., Vergin K.L., Landry Z.C., Ellisman M.H., Deerinck T.J., Sullivan M.B., Giovannoni S.J. (2013). Abundant SAR11 viruses in the ocean. Nat. Cell Biol..

[47] Antunes A., Alam I., Simões M.F., Daniels C.A., Ferreira A.J., Siam R., El-Dorry H., Bajic V.B. (2015). First Insights into the Viral Communities of the Deep-sea Anoxic Brines of the Red Sea. Genom. Proteom. Bioinform..

[48] Jasna V., Ammini P., Dash A. (2018). Genetic and functional diversity of double-stranded DNA viruses in a tropical monsoonal estuary, India. Sci. Rep..

[49] Janelidze N., Jaiani E., Lashkhi N., Tskhvediani A., Kokashvili T., Gvarishvili T., Jgenti D., Mikashavidze E., Diasamidze R., Narodny S. (2011). Microbial water quality of the Georgian coastal zone of the Black Sea. Mar. Pollut. Bull..

[50] Paul J.H. (2008). Prophages in marine bacteria: Dangerous molecular time bombs or the key to survival in the seas?. ISME J..

[51] McDaniel L., Houchin L.A., Williamson S.J., Paul J.H. (2002). Lysogeny in marine Synechococcus. Nat. Cell Biol..

[52] Ortmann A., Lawrence J., Suttle C. (2002). Lysogeny and Lytic Viral Production during a Bloom of the Cyanobacterium Synechococcus spp. Microb. Ecol..

[53] Zhao Y., Qin F., Zhang R., Giovannoni S.J., Zhang Z., Sun J., Du S., Rensing C. (2018). Pelagiphages in the Podoviridae family integrate into host genomes. Environ. Microbiol..

[54] Zaremba-Niedzwiedzka K., Viklund J., Zhao W., Ast J., Sczyrba A., Woyke T., McMahon K.D., Bertilsson S., Stepanauskas R., Andersson S.G. (2013). Single-cell genomics reveal low recombination frequencies in freshwater bacteria of the SAR11 clade. Genome Biol..

[55] Seed K.D., Lazinski D.W., Calderwood S.B., Camilli A. (2013). A bacteriophage encodes its own CRISPR/Cas adaptive response to evade host innate immunity. Nat. Cell Biol..

[56] Pausch P., Al-Shayeb B., Bisom-Rapp E. (2020). Crispr-casf from huge phages is a hypercompact genome editor. Science.

[57] Jiang S.C., Paul J.H. (1998). Gene Transfer by Transduction in the Marine Environment. Appl. Environ. Microbiol..

[58] Koonin E.V., Makarova K.S., Aravind L. (2001). Horizontal Gene Transfer in Prokaryotes: Quantification and Classification. Annu. Rev. Microbiol..

[59] Sano E., Carlson S., Wegley L., Rohwer F. (2004). Movement of Viruses between Biomes. Appl. Environ. Microbiol..

[60] Majkowska-Skrobek G., Maciejewska B. (2015). Bacteriophages and Phage-Derived Proteins–Application Approaches. Curr. Med. Chem..

[61] Yamaguchi H., Furuhata K., Fukushima T., Yamamoto H., Sekiguchi J. (2004). Characterization of a new Bacillus subtilis peptidoglycan hydrolase gene, yvcE (named cwlO), and the enzymatic properties of its encoded protein. J. Biosci. Bioeng..

[62] John S.G., Mendez C.B., Deng L., Poulos B., Kauffman A.K.M., Kern S., Brum J., Polz M.F., Boyle E.A., Sullivan M.B. (2011). A Simple and Efficient Method for Concentration of Ocean Viruses by Chemical Flocculation. Environ. Microbiol. Rep..

[63] Chen I.-M., Chu K., Palaniappan K., Pillay M., Ratner A., Huang J., Huntemann M., Varghese N., White J.R., Seshadri R. (2019). IMG/M v.5.0: An integrated data management and comparative analysis system for microbial genomes and microbiomes. Nucleic Acids Res..

[64] Arndt D., Grant J.R., Marcu A., Sajed T., Pon A., Liang Y., Wishart D.S. (2016). PHASTER: A better, faster version of the PHAST phage search tool. Nucleic Acids Res..

[65] Li P.-E., Lo C.-C., Anderson J.J., Davenport K.W., Bishop-Lilly K.A., Xu Y., Ahmed S., Feng S., Mokashi V.P., Chain P.S. (2016). Enabling the democratization of the genomics revolution with a fully integrated web-based bioinformatics platform. Nucleic Acids Res..

[66] Wood D., Salzberg S.L. (2014). Kraken: Ultrafast metagenomic sequence classification using exact alignments. Genome Biol..

[67] Meyer F., Paarmann D., Souza M.D., Olson R., Glass E.M., Kubal M., Paczian T., Rodriguez A., Stevens R., Wilke A. (2008). The metagenomics RAST server—A public resource for the automatic phylogenetic and functional analysis of metagenomes. BMC Bioinform..

