# Supplementary material for: Microbial Diversity and Phage–Host Interactions in the Georgian Coastal Area of the Black Sea Revealed by Whole Genome Metagenomic Sequencing
Source: Mar Drugs. 2020 Nov 14;18(11):558. doi: 10.3390/md18110558 (PMC7697616; doi:10.3390/md18110558)
Supplement: Supplementary file 1 [file marinedrugs-18-00558-s001.zip › marinedrugs-977417-SI/Table S1.docx]

Table S1: General characteristics of the Black Sea metagenomes

| Sample | Black Sea Area | Date of sampling | Biome type | Number of Reads | Number of Trimmed Reads | Number of Contigs | Min length of contig (bp) |
| --- | --- | --- | --- | --- | --- | --- | --- |
| P21 | Poti | May,  2018 | Prokaryotes | 2 353 175 | 2 228 316 | 1830 | 1000 |
|  |  |  |  | 2 353 175 | 2 222 432 |  |  |
| P24 | Gonio | May,  2018 | Prokaryotes | 2 197 729 | 1 125 523 | 286 | 1000 |
|  |  |  |  | 2 197 729 | 2027 751 |  |  |
| P31 | Poti | September,  2018 | Prokaryotes | 2 638 018 | 1518830 | 2752 | 1000 |
|  |  |  |  | 2 638 018 | 1895710 |  |  |
| P34 | Gonio | September,  2018 | Prokaryotes | 3 836 608 | 2 955 016 | 2233 | 1000 |
|  |  |  |  | 3 836 608 | 2393060 |  |  |
| V26 | Poti | May, 2018 | Viruses | 6 309 194 | 5 625 988 | 7286 | 1000 |
|  |  |  |  | 6 309 194 | 5 439 257 |  |  |
| V29 | Gonio | May, 2018 | Viruses | 5 953 337 | 5 144 155 | 8167 | 1000 |
|  |  |  |  | 5 953 337 | 4 936 673 |  |  |
| V34 | Gonio | September,  2018 | Viruses | 3 134 657 | 2 955 016 | 2233 | 1000 |
|  |  |  |  | 3 134 657 | 2393060 |  |  |
| V36 | Poti | September,  2018 | Viruses | 3 020 702 | 2964 436 | 4678 | 1000 |
|  |  |  |  | 3 020 702 | 2958 702 |  |  |

.
