# Supplementary material for: Microbial Diversity and Phage–Host Interactions in the Georgian Coastal Area of the Black Sea Revealed by Whole Genome Metagenomic Sequencing
Source: Mar Drugs. 2020 Nov 14;18(11):558. doi: 10.3390/md18110558 (PMC7697616; doi:10.3390/md18110558)
Supplement: Supplementary file 1 [file marinedrugs-18-00558-s001.zip › marinedrugs-977417-SI/Table S3.docx]

| **Table S3. Putative prophages in the Black Sea prokaryotic contigs** | | | |
| --- | --- | --- | --- |
| **Similar prophages** | ***Sequence length in kbp*** | ***Number of similar proteins*** | ***Sampling location*** |
| PHAGE_Synech_syn9_NC_008296 | 16.9 | 18 | Poti September 2018 |
| PHAGE_Synech_S_SM2_NC_015279 | 14.2 | 22 | Poti September 2018 |
| PHAGE_Synech_syn9_NC_008296 | 9.4 | 17 | Poti September 2018 |
| PHAGE_Synech_S_SKS1_NC_020851 | 9.4 | 17 | Poti September 2018 |
| PHAGE_Synech_S_WAM1_NC_031944 | 9.1 | 17 | Poti September 2018 |
| PHAGE_Synech_A.HR1_NC_020486 | 6.1 | 22 | Poti September 2018 |
| PHAGE_Synech_S_SM2_NC_015279 | 7.6 | 20 | Poti September 2018 |
| PHAGE_Synech_syn9_NC_008296 | 8.5 | 21 | Poti September 2018 |
| PHAGE_Synech_S_SSM7_NC_015287 | 42.3 | 37 | Gonio September 2018 |
| PHAGE_Synech_S_SM2_NC_015279 | 17.1 | 36 | Gonio September 2018 |
| PHAGE_Synech_S_SKS1_NC_020851 | 30.6 | 46 | Gonio September 2018 |
| PHAGE_Synech_S_SKS1_NC_020851 | 23.2 | 56 | Gonio September 2018 |
| PHAGE_Agroba_Atu_ph07_NC_042013 | 10.1 | 16 | Gonio September 2018 |
