# Supplementary material for: Microbial Diversity and Phage–Host Interactions in the Georgian Coastal Area of the Black Sea Revealed by Whole Genome Metagenomic Sequencing
Source: Mar Drugs. 2020 Nov 14;18(11):558. doi: 10.3390/md18110558 (PMC7697616; doi:10.3390/md18110558)
Supplement: Supplementary file 1 [file marinedrugs-18-00558-s001.zip › marinedrugs-977417-SI/Table S4.docx]

**Table S4. CRISPR arrays in the Black Sea prokaryotic and viral metagenomes**

*Poti May 2018 viral metagenome*

CRISPR sequences found in *Flavobacteriaceae*:

| CRISPR Number | Start Coordinate | End Coordinate | Position | Repeat Sequence | Spacer Sequence |
| --- | --- | --- | --- | --- | --- |
| 1 | 778 | 904 | 778 | TACAACAGGATGTTCTAACGTTGC | TATAGGTTATAATTCATTATACAATAA |
| 1 | 778 | 904 | 829 | TACAACAGGTGTTAATAACATTGC | TATAGGAACATGTGCTTATTGTTCTGG |
| 1 | 778 | 904 | 880 | TACAACAGGTATTAATAACATTGC |  |

CRISPR sequences found in *Pelagibacteriaceae*:

| CRISPR Number | Start Coordinate | End Coordinate | Position | Repeat Sequence | Spacer Sequence |
| --- | --- | --- | --- | --- | --- |
| 1 | 1838 | 1970 | 1838 | AAAAAAACTACCAAAAAGAAA | ATTGTCAAAGCGAAAGCTGTAAAGAAAGCTGCAGTA |
| 1 | 1838 | 1970 | 1895 | AAAAAAACAACCACAAAGAAA | AAAACTGTTACTAAAAAAGTAGCAAAAGTTACA |
| 1 | 1838 | 1970 | 1949 | AAAAAAACTGCAAAGAAAGTA |  |

*Poti May 2018 prokaryotic metagenome*

CRISPR sequences could not be assigned to any known microbial sequences in NCBI:

| CRISPR Number | Start Coordinate | End Coordinate | Position | Repeat Sequence | Spacer Sequence |
| --- | --- | --- | --- | --- | --- |
| 1 | 1161 | 1308 | 1161 | AGGAATCAGGAATCAGGAATCA | GGAATCAGGAATCAGAATCCCGGTCAAAATGTTGGAATC |
| 1 | 1161 | 1308 | 1222 | AGGAATCAGGAATCAGGAATCA | GGAATCAGAATCCCGGTCAAAATGTGGGCGATCCGACAAATC |
| 1 | 1161 | 1308 | 1286 | AGGAATCAGGAATCAGGAATCA |  |

*Gonio May 2018 viral metagenome*

CRISPR sequences could not be assigned to any known microbial sequences in NCBI:

| CRISPR Number | Start Coordinate | End Coordinate | Position | Repeat Sequence | Spacer Sequence |
| --- | --- | --- | --- | --- | --- |
| 1 | 1837 | 2308 | 1837 | ACACTACTTATATTCCAATTGTCAATATCTTGATTAAAT | GAACTATTTTCAAACATTAAAATCATCGTTCTT |
| 1 | 1837 | 2308 | 1909 | ACATTAGATACATCCCAATTTCCAATAAATTGATTGAAT | TTACTATCACGAAACATGTAATTCATATTAACT |
| 1 | 1837 | 2308 | 1981 | ACATTGGATACATCCCAATTTCCAATAGGTTGATTGAAT | TCACTTTCTAAAAACATATGTTTCATATCTTCA |
| 1 | 1837 | 2308 | 2053 | ACATTCGATACATCCCAATTTCCAATGTCTTGATTAAAT | ATAGATCGGTCAAACATAGAACTCATAGTTTCA |
| 1 | 1837 | 2308 | 2125 | ACATTAGATACATCCCAAAGTCCAATATATTGATTGAAT | AAACTGAAACCAAACATATTACTCATGTTAATT |
| 1 | 1837 | 2308 | 2197 | ACATTAGATACATCCCAATAAGCAATATTCTTATCAAAT | TTTGAATTATAAAACATATGATTCATATAAATG |
| 1 | 1837 | 2308 | 2269 | ACATTCGAAACATCCCAATTTCCAATGTGTTGATTAAAT |  |

CRISPR sequences could not be assigned to any known microbial sequences in NCBI:

| CRISPR Number | Start Coordinate | End Coordinate | Position | Repeat Sequence | Spacer Sequence |
| --- | --- | --- | --- | --- | --- |
| 1 | 1 | 168 | 1 | CCGAGAATTGC | ACGTCGCTTACTCATCTTAACATGTCTCACAACGAGCA |
| 1 | 1 | 168 | 50 | AGTGACTGATGCTGGTGTCATCGCCATTGCCGAGAATTGC | GAATTGCTTACTCATCTTGACATGACTAACTGCGGCAG |
| 1 | 1 | 168 | 128 | AGTGACTGATGCTGGTATCATCGCCATTGCCGAGAATTGC |  |

*Gonio September 2018 prokaryotic metagenome*

CRISPR sequences could not be assigned to any known microbial sequences in NCBI:

| CRISPR Number | Start Coordinate | End Coordinate | Position | Repeat Sequence | Spacer Sequence |
| --- | --- | --- | --- | --- | --- |
| 1 | 480 | 609 | 480 | GCAGGACGGGACATTCCCTGCCGC | TGCCTCCCAGGATTCGAACCCGAGGCGCT |
| 1 | 480 | 609 | 533 | GCAGGACGGGCCATTCCCTGCCGC | CATTTTCGGGATTCGAACCAGCGGCGCT |
| 1 | 480 | 609 | 585 | GTAGGACGGGTCATTCCCTACCGC |  |

CRISPR sequences assigned to unclassified Sphingomonadales:

| **CRISPR Number** | **Start Coordinate** | **End Coordinate** | **Position** | **Repeat Sequence** | **Spacer Sequence** |
| --- | --- | --- | --- | --- | --- |
| 1 | 1 | 183 | 1 | AGAAAGAGAGAGAGAGA | AAATAAAGAGAGAAAAGAAAGAAAAAGAGAGAAAGAGATGTGTGTGTGTGTGTGAGA |
| 1 | 1 | 183 | 75 | GAGAGAGAGAGAGAAAGAGAGAGAGAGA | GAGAGAGAGAGAGAGAGAGAGAGAGAGAGAGAGAGAGAGAGAGAGAGAGAAG |
| 1 | 1 | 183 | 155 | GAGAGAGAGAGAGAAAGAGAGAGAAAGA |  |

CRISPR sequences could not be assigned to any known microbial sequences in NCBI:

| CRISPR Number | Start Coordinate | End Coordinate | Position | Repeat Sequence | Spacer Sequence |
| --- | --- | --- | --- | --- | --- |
| 1 | 1382 | 1557 | 1382 | TTTTTCAACCGTCGAAAATGCGCATTT | TTTAATGCGCATTTACAGCCAATTTTGAGGCATTTGGAATTAAGGGACT |
| 1 | 1382 | 1557 | 1458 | TTTATAAAACGCATTTATTGCGCATTT | AATGCGCACTTTTTAATGCGCATTTTTTCAGCATTTTTTCGGCAT |
| 1 | 1382 | 1557 | 1530 | TTTTTAAAACGCATTTAATGCGCATTT |  |

*Gonio September 2018 prokaryotic metagenome*

CRISPR sequences assigned to uncultured *Myoviruidae* phage MedDCM-OCT-S09-C7:

| CRISPR Number | Start Coordinate | End Coordinate | Position | Repeat Sequence | Spacer Sequence |
| --- | --- | --- | --- | --- | --- |
| 1 | 27180 | 27333 | 27180 | CTAATATTAGGTTGTTGATAATTAACAGGATTT | TGATAAATTGCTGGTTGTCTTGCATCT |
| 1 | 27180 | 27333 | 27240 | CTAATGTTAGGTTCTTGGTAGTTTACAGGATTT | CTATAGGTTGCAGGTTGTCTTGCATTC |
| 1 | 27180 | 27333 | 27300 | CTAATATTAGGTTGTTGATAGTTTACAGGATTT |  |

CRISPR sequences assigned to *Microcystaceae*:

| CRISPR Number | Start Coordinate | End Coordinate | Position | Repeat Sequence | Spacer Sequence |
| --- | --- | --- | --- | --- | --- |
| 1 | 2038 | 2620 | 2038 | CTCCGAGTCCCTCCTAAAAAA | TATAGCGCTTGAAGTTTTTGCCTGACGCTCCGGGCTTAAAACGTC |
| 1 | 2038 | 2620 | 2104 | CTCCGAGCACCTCTTAAAAAA | TCTCCGACTCGAAAGAAAAACTACTATGCGCAACCCGGTGTGGCACAACGA |
| 1 | 2038 | 2620 | 2176 | CTCCGAGCACCTCTTAAAAAA | ATCTCCGACTCGAAGTGTCTTTCAATAAAATTGCATCTTTGTAAAAAT |
| 1 | 2038 | 2620 | 2245 | CTCCGAGTCCCTCTTAAAAAA | TCTACCACTCGAAGCGTTTGTCATCCTGTTAGCTAAACGGTTGGTAAGG |
| 1 | 2038 | 2620 | 2315 | CTCCGAGTTCTTCCTAAAAAA | TCTAGCGCTCGAAGGGTATCGGGCGCAACCGTTGACCTGAAAAACTACTA |
| 1 | 2038 | 2620 | 2386 | CTCCGAGTCCTTCCCAAAATA | ATCTACCTCTCGAAGCCCGACTCTTCAGGGCAATATACACTTACAACAGAT |
| 1 | 2038 | 2620 | 2458 | CTCCGAGTCCCTCTCAAAATA | ATCTACCTCTCGAAGTAAAAAATTAGATATTAGGCTCCCGTCCGTAGCAT |
| 1 | 2038 | 2620 | 2529 | CTCCGAGTCCCTCTCAAAATA | ATTTACCACTCGAAGACAATGATAAGCGAACGCCCTCTTTAATCAGCGC |
| 1 | 2038 | 2620 | 2599 | CTCCGAGTCCCTCTCAAAATA |  |

CRISPR sequences assigned to Marine virus AFVG:

| CRISPR Number | Start Coordinate | End Coordinate | Position | Repeat Sequence | Spacer Sequence |
| --- | --- | --- | --- | --- | --- |
| 1 | 486 | 636 | 486 | TGTACCTTGTGTTGATGTAAT | AGATAGACCTGTAGGCTCAACTAACGTTAAAGTTTCAACAGTTGG |
| 1 | 486 | 636 | 552 | TGTACCTTGTGCTGATGTAAT | TTCAAAACCTGTAACCGATACATCTTGGTTTGGTGCAACCGC |
| 1 | 486 | 636 | 615 | TGTGCCTTGTGCTGATGTAAT |  |

CRISPR sequences assigned to uncultured Caudovirales phage:

| CRISPR Number | Start Coordinate | End Coordinate | Position | Repeat Sequence | Spacer Sequence |
| --- | --- | --- | --- | --- | --- |
| 1 | 787 | 938 | 787 | ACCCTCTCACGAACCCCTCCGGCTACATTAC | GTCCTCGGCGCTGACTGGCTACGCCACGG |
| 1 | 787 | 938 | 847 | AGTCCTTCGTCACGTCTCAAGGCTACATCAC | CGCATCCGCCCTGACAGGCTATGCGACGG |
| 1 | 787 | 938 | 907 | AGTCCTTCGTCACGTCTCAGGGCTACATCAC |  |
