# Supplementary material for: Microbial Diversity and Phage–Host Interactions in the Georgian Coastal Area of the Black Sea Revealed by Whole Genome Metagenomic Sequencing
Source: Mar Drugs. 2020 Nov 14;18(11):558. doi: 10.3390/md18110558 (PMC7697616; doi:10.3390/md18110558)
Supplement: Supplementary file 1 [file marinedrugs-18-00558-s001.zip › marinedrugs-977417-SI/Table S6.docx]

| Microorganism  **Table S6**. Occurrence of peptidoglycan hydrolase genes in the Black Sea metagenomes | Start coord | | End  coord | | Gene length | scaffold | Scaffold length | COG ID | | COG Function | | The Black Sea site and sampling time | |
| --- | --- | --- | --- | --- | --- | --- | --- | --- | --- | --- | --- | --- | --- |
| Bacteria;Bacteroidetes;Flavobacteriia;Flavobacteriales;Flavobacteriaceae | 4304 | | 5137 | | 834 | [Ga0392065_1666](https://img.jgi.doe.gov/cgi-bin/mer/main.cgi?section=MetaScaffoldDetail&page=metaScaffoldDetail&scaffold_oid=Ga0392065_1666&taxon_oid=3300035430&data_type=assembled) | [19958](https://img.jgi.doe.gov/cgi-bin/mer/main.cgi?section=MetaScaffoldGraph&page=metaScaffoldGraph&scaffold_oid=Ga0392065_1666&taxon_oid=3300035430&data_type=assembled&start_coord=1&end_coord=19958&marker_gene=Ga0392065_1666_4304_5137&seq_length=19958) | COG1705 | | Flagellum-specific peptidoglycan hydrolase FlgJ | | Poti May 2018 | |
| Viruses; unclassified viruses; unclassified bacterial viruses; Marine virus AFVG | 2993 | | 3334 | | 342 | [Ga0392065_1979](https://img.jgi.doe.gov/cgi-bin/mer/main.cgi?section=MetaScaffoldDetail&page=metaScaffoldDetail&scaffold_oid=Ga0392065_1979&taxon_oid=3300035430&data_type=assembled) | [6623](https://img.jgi.doe.gov/cgi-bin/mer/main.cgi?section=MetaScaffoldGraph&page=metaScaffoldGraph&scaffold_oid=Ga0392065_1979&taxon_oid=3300035430&data_type=assembled&start_coord=1&end_coord=6623&marker_gene=Ga0392065_1979_2993_3334&seq_length=6623) | COG3883 | | Uncharacterized N-terminal domain of peptidoglycan hydrolase CwlO | | Poti May 2018 | |
| Viruses; Duplodnaviria; Heunggongvirae; Uroviricota; Caudoviricetes;Caudovirales; Myoviridae; Pelagibacter phage | 61 | | 315 | | 255 | [Ga0392065_3038](https://img.jgi.doe.gov/cgi-bin/mer/main.cgi?section=MetaScaffoldDetail&page=metaScaffoldDetail&scaffold_oid=Ga0392065_3038&taxon_oid=3300035430&data_type=assembled) | [2713](https://img.jgi.doe.gov/cgi-bin/mer/main.cgi?section=MetaScaffoldGraph&page=metaScaffoldGraph&scaffold_oid=Ga0392065_3038&taxon_oid=3300035430&data_type=assembled&start_coord=1&end_coord=2713&marker_gene=Ga0392065_3038_61_315&seq_length=2713) | COG3883 | | Uncharacterized N-terminal domain of peptidoglycan hydrolase CwlO | | Poti May 2018 | |
| Viruses; Duplodnaviria; Heunggongvirae; Uroviricota; Caudoviricetes;Caudovirales; Myoviridae; Pelagibacter phage | 238 | | 549 | | 312 | [Ga0392065_3722](https://img.jgi.doe.gov/cgi-bin/mer/main.cgi?section=MetaScaffoldDetail&page=metaScaffoldDetail&scaffold_oid=Ga0392065_3722&taxon_oid=3300035430&data_type=assembled) | [1384](https://img.jgi.doe.gov/cgi-bin/mer/main.cgi?section=MetaScaffoldGraph&page=metaScaffoldGraph&scaffold_oid=Ga0392065_3722&taxon_oid=3300035430&data_type=assembled&start_coord=1&end_coord=1384&marker_gene=Ga0392065_3722_238_549&seq_length=1384) | COG3883 | | Uncharacterized N-terminal domain of peptidoglycan hydrolase CwlO | | Poti May 2018 | |
| Bacteria;Proteobacteria;Alphaproteobacteria;Pelagibacterales;Pelagibacteraceae;Candidatus Pelagibacter;Candidatus Pelagibacter ubique | 3 | | 314 | | 312 | [Ga0392065_4399](https://img.jgi.doe.gov/cgi-bin/mer/main.cgi?section=MetaScaffoldDetail&page=metaScaffoldDetail&scaffold_oid=Ga0392065_4399&taxon_oid=3300035430&data_type=assembled) | [2973](https://img.jgi.doe.gov/cgi-bin/mer/main.cgi?section=MetaScaffoldGraph&page=metaScaffoldGraph&scaffold_oid=Ga0392065_4399&taxon_oid=3300035430&data_type=assembled&start_coord=1&end_coord=2973&marker_gene=Ga0392065_4399_3_314&seq_length=2973) | COG0797 | | Rare lipoprotein A, peptidoglycan hydrolase digesting "naked" glycans, contains C-terminal SPOR domain | | Poti May 2018 | |
| Viruses; Duplodnaviria; Heunggongvirae; Uroviricota; Caudoviricetes; Caudovirales; Siphoviridae;Uncultured Mediterranean phage uvMED | 1523 | | 1738 | | 216 | [Ga0392065_5204](https://img.jgi.doe.gov/cgi-bin/mer/main.cgi?section=MetaScaffoldDetail&page=metaScaffoldDetail&scaffold_oid=Ga0392065_5204&taxon_oid=3300035430&data_type=assembled) | [2335](https://img.jgi.doe.gov/cgi-bin/mer/main.cgi?section=MetaScaffoldGraph&page=metaScaffoldGraph&scaffold_oid=Ga0392065_5204&taxon_oid=3300035430&data_type=assembled&start_coord=1&end_coord=2335&marker_gene=Ga0392065_5204_1523_1738&seq_length=2335) | COG3883 | | Uncharacterized N-terminal domain of peptidoglycan hydrolase CwlO | | Poti May 2018 | |
| Uncultured Mediterranean phage clone uvDeep-GF1-KM14-C251 | 1495 | | 1779 | | 285 | [Ga0392065_6386](https://img.jgi.doe.gov/cgi-bin/mer/main.cgi?section=MetaScaffoldDetail&page=metaScaffoldDetail&scaffold_oid=Ga0392065_6386&taxon_oid=3300035430&data_type=assembled) | [2332](https://img.jgi.doe.gov/cgi-bin/mer/main.cgi?section=MetaScaffoldGraph&page=metaScaffoldGraph&scaffold_oid=Ga0392065_6386&taxon_oid=3300035430&data_type=assembled&start_coord=1&end_coord=2332&marker_gene=Ga0392065_6386_1495_1779&seq_length=2332) | COG3883 | | Uncharacterized N-terminal domain of peptidoglycan hydrolase CwlO | | Poti May 2018 | |
| Viruses; Duplodnaviria; Heunggongvirae; Uroviricota; Caudoviricetes;Caudovirales; Myoviridae.  Pelagibacter phage | 5443 | | 5694 | | 252 | [Ga0392065_6500](https://img.jgi.doe.gov/cgi-bin/mer/main.cgi?section=MetaScaffoldDetail&page=metaScaffoldDetail&scaffold_oid=Ga0392065_6500&taxon_oid=3300035430&data_type=assembled) | [5705](https://img.jgi.doe.gov/cgi-bin/mer/main.cgi?section=MetaScaffoldGraph&page=metaScaffoldGraph&scaffold_oid=Ga0392065_6500&taxon_oid=3300035430&data_type=assembled&start_coord=1&end_coord=5705&marker_gene=Ga0392065_6500_5443_5694&seq_length=5705) | COG1705 | | Flagellum-specific peptidoglycan hydrolase FlgJ | | Poti May 2018 | |
| Viruses; Duplodnaviria; Heunggongvirae; Uroviricota; Caudoviricetes;Caudovirales; Myoviridae.  Pelagibacter phage | 712 | | 975 | | 264 | [Ga0392065_7071](https://img.jgi.doe.gov/cgi-bin/mer/main.cgi?section=MetaScaffoldDetail&page=metaScaffoldDetail&scaffold_oid=Ga0392065_7071&taxon_oid=3300035430&data_type=assembled) | [1438](https://img.jgi.doe.gov/cgi-bin/mer/main.cgi?section=MetaScaffoldGraph&page=metaScaffoldGraph&scaffold_oid=Ga0392065_7071&taxon_oid=3300035430&data_type=assembled&start_coord=1&end_coord=1438&marker_gene=Ga0392065_7071_712_975&seq_length=1438) | COG3883 | | Uncharacterized N-terminal domain of peptidoglycan hydrolase CwlO | | Poti May 2018 | |
| Bacteria; Bacteroidetes; Flavobacteriia;Flavobacteriales; Flavobacteriaceae; Aquimarina; unclassified Aquimarina AD10 | | 11072 | 11872 | 801 | | [Ga0449474_0381](https://img.jgi.doe.gov/cgi-bin/mer/main.cgi?section=MetaScaffoldDetail&page=metaScaffoldDetail&scaffold_oid=Ga0449474_0381&taxon_oid=3300040768&data_type=assembled) | [19732](https://img.jgi.doe.gov/cgi-bin/mer/main.cgi?section=MetaScaffoldGraph&page=metaScaffoldGraph&scaffold_oid=Ga0449474_0381&taxon_oid=3300040768&data_type=assembled&start_coord=1&end_coord=19732&marker_gene=Ga0449474_0381_11072_11872&seq_length=19732) | COG1705 | Flagellum-specific peptidoglycan hydrolase FlgJ | | Poti May 2018 | |  |
| Viruses; unclassified viruses; unclassified bacterial viruses; Marine virus AFVG_25M178 | | 5750 | 6208 | 459 | | [Ga0449472_2316](https://img.jgi.doe.gov/cgi-bin/mer/main.cgi?section=MetaScaffoldDetail&page=metaScaffoldDetail&scaffold_oid=Ga0449472_2316&taxon_oid=3300040766&data_type=assembled) | [6231](https://img.jgi.doe.gov/cgi-bin/mer/main.cgi?section=MetaScaffoldGraph&page=metaScaffoldGraph&scaffold_oid=Ga0449472_2316&taxon_oid=3300040766&data_type=assembled&start_coord=1&end_coord=6231&marker_gene=Ga0449472_2316_5750_6208&seq_length=6231) | COG0797 | Rare lipoprotein A, peptidoglycan hydrolase digesting "naked" glycans, contains C-terminal SPOR domain | | Gonio May  2018 | |  |
| Bacteria; Proteobacteria;Alphaproteobacteria; Pelagibacterales; Pelagibacteraceae; Candidatus Pelagibacter; unclassified Candidatus Pelagibacter | | 592 | 915 | 324 | | [Ga0449472_6686](https://img.jgi.doe.gov/cgi-bin/mer/main.cgi?section=MetaScaffoldDetail&page=metaScaffoldDetail&scaffold_oid=Ga0449472_6686&taxon_oid=3300040766&data_type=assembled) | [1800](https://img.jgi.doe.gov/cgi-bin/mer/main.cgi?section=MetaScaffoldGraph&page=metaScaffoldGraph&scaffold_oid=Ga0449472_6686&taxon_oid=3300040766&data_type=assembled&start_coord=1&end_coord=1800&marker_gene=Ga0449472_6686_592_915&seq_length=1800) | COG3883 | Uncharacterized N-terminal domain of peptidoglycan hydrolase CwlO | | Gonio May  2018 | |  |
| Bacteria; Proteobacteria;Alphaproteobacteria; Pelagibacterales; Pelagibacteraceae; Candidatus Pelagibacter; unclassified Candidatus Pelagibacter | | 268 | 987 | 720 | | [Ga0449472_7917](https://img.jgi.doe.gov/cgi-bin/mer/main.cgi?section=MetaScaffoldDetail&page=metaScaffoldDetail&scaffold_oid=Ga0449472_7917&taxon_oid=3300040766&data_type=assembled) | [1087](https://img.jgi.doe.gov/cgi-bin/mer/main.cgi?section=MetaScaffoldGraph&page=metaScaffoldGraph&scaffold_oid=Ga0449472_7917&taxon_oid=3300040766&data_type=assembled&start_coord=1&end_coord=1087&marker_gene=Ga0449472_7917_268_987&seq_length=1087) | COG0797 | Rare lipoprotein A, peptidoglycan hydrolase digesting "naked" glycans, contains C-terminal SPOR domain | | Gonio May 2018 | |  |
